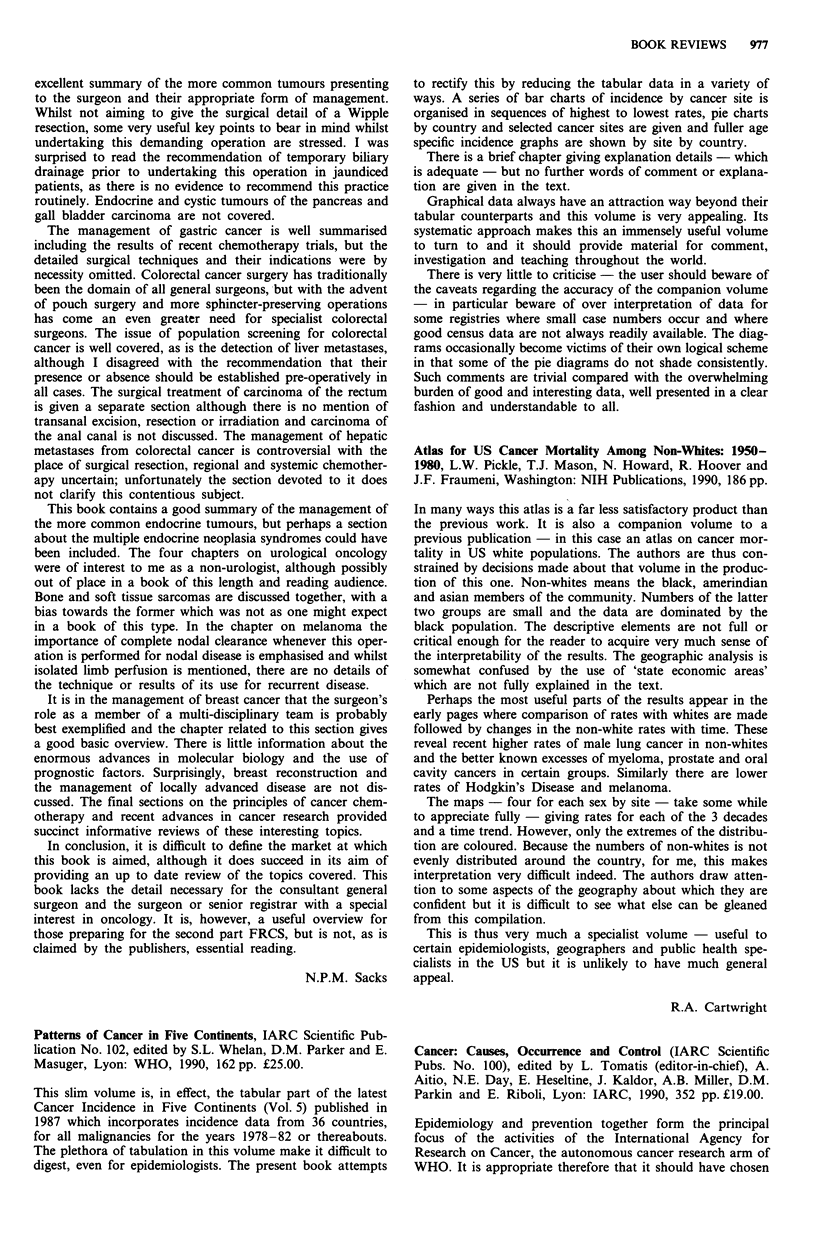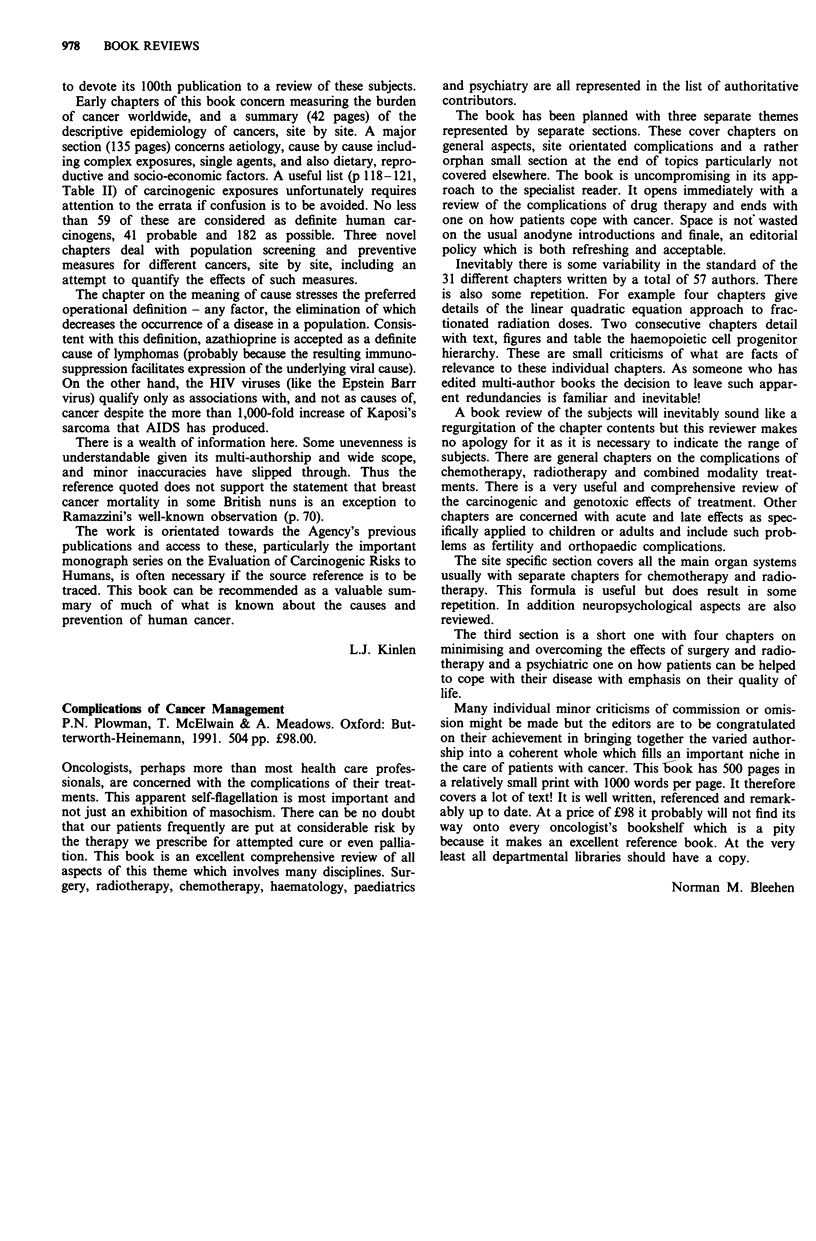# Cancer: Causes, Occurrence and Control

**Published:** 1991-11

**Authors:** L.J. Kinlen


**Cancer: Causes, Occurrence and Control** (IARC Scientific Pubs. No. 100), edited by L. Tomatis (editor-in-chief), A. Aitio, N.E. Day, E. Heseltine, J. Kaldor, A.B. Miller, D.M. Parkin and E. Riboli, Lyon: IARC, 1990, 352 pp. £19.00.

Epidemiology and prevention together form the principal focus of the activities of the International Agency for Research on Cancer, the autonomous cancer research arm of WHO. It is appropriate therefore that it should have chosen to devote its 100th publication to a review of these subjects.

Early chapters of this book concern measuring the burden of cancer worldwide, and a summary (42 pages) of the descriptive epidemiology of cancers, site by site. A major section (135 pages) concerns aetiology, cause by cause including complex exposures, single agents, and also dietary, reproductive and socio-economic factors. A useful list (p 118–121, Table II) of carcinogenic exposures unfortunately requires attention to the errata if confusion is to be avoided. No less than 59 of these are considered as definite human carcinogens, 41 probable and 182 as possible. Three novel chapters deal with population screening and preventive measures for different cancers, site by site, including an attempt to quantify the effects of such measures.

The chapter on the meaning of cause stresses the preferred operational definition – any factor, the elimination of which decreases the occurrence of a disease in a population. Consistent with this definition, azathioprine is accepted as a definite cause of lymphomas (probably because the resulting immunosuppression facilitates expression of the underlying viral cause). On the other hand, the HIV viruses (like the Epstein Barr virus) qualify only as associations with, and not as causes of, cancer despite the more than 1,000-fold increase of Kaposi's sarcoma that AIDS has produced.

There is a wealth of information here. Some unevenness is understandable given its multi-authorship and wide scope, and minor inaccuracies have slipped through. Thus the reference quoted does not support the statement that breast cancer mortality in some British nuns is an exception to Ramazzini's well-known observation (p. 70).

The work is orientated towards the Agency's previous publications and access to these, particularly the important monograph series on the Evaluation of Carcinogenic Risks to Humans, is often necessary if the source reference is to be traced. This book can be recommended as a valuable summary of much of what is known about the causes and prevention of human cancer.

L.J. Kinlen